# Geniposide, a Principal Component of Gardeniae Fructus, Protects Skin from Diesel Exhaust Particulate Matter-Induced Oxidative Damage

**DOI:** 10.1155/2021/8847358

**Published:** 2021-02-02

**Authors:** Ly Thi Huong Nguyen, Sang Hyun Ahn, Uy Thai Nguyen, In Jun Yang, Heung Mook Shin

**Affiliations:** ^1^Department of Physiology, College of Korean Medicine, Dongguk University, Gyeongju 38066, Republic of Korea; ^2^Department of Anatomy, College of Korean Medicine, Semyung University, Jecheon-si 27136, Republic of Korea

## Abstract

Gardeniae Fructus (GF) is the fruit of *Gardenia jasminoides* Ellis and is traditionally prescribed to treat pyogenic infections and skin ulcers. This study investigated the protective effects of GF and the underlying mechanism responsible for these effects on diesel exhaust particulate matter- (DEP-) induced skin damage. The protective effects of an ethanolic extract of GF (GFE) and its constituents (geniposidic acid, gardenoside, geniposide, chlorogenic acid, and genipin) were examined by analyzing reactive oxygen species (ROS) production, apoptosis, and tight junction (TJ) protein expression in HaCaT cells. Treatment with GFE dose-dependently inhibited intracellular ROS production and apoptosis by regulating the protein expressions of Bax, Bcl-2, and cytochrome C in DEP-stimulated (100 *μ*g/ml) HaCaT cells. Mechanistic studies revealed that the protective effects of GFE were related to its activation of Nrf2 and HO-1 signaling in HaCaT cells. Geniposide, a main constituent of GFE, enhanced the expression of occludin in DEP-stimulated HaCaT cells. Furthermore, topical application of geniposide reduced the expressions of 8-OHdG and Bax and increased the expression of occludin in the dorsal skin lesions of DEP-stimulated mice. Gardeniae Fructus and its main component geniposide are potential candidates for the repair of DEP-induced skin damage due to their antioxidant and antiapoptotic activities.

## 1. Introduction

Air pollution is cited as a major risk factor of skin aging as it promotes pigmentation and wrinkle formation [1, 2]. Air pollution causes or aggravates inflammatory skin conditions such as atopic dermatitis, acne, and psoriasis [3], and as a result, antipollution cosmetics are being actively developed. Furthermore, it has been established that these types of products protect the skin from oxidative stress and inflammation-induced damage [4, 5]. The main air pollutant is particulate matter (PM), that is, the sum of all solid and liquid particles arising from fuel combustion and road traffic. According to a recent study, airborne PM with diameters <1 micron remains in the lungs a week after inhalation [6]. PM can also directly penetrate skin, because skin pores are larger than PM diameters, and thus, PM penetrates skin directly through hair follicles and damages skin barrier integrity, which facilitates further penetration [7, 8].

Gardeniae Fructus (GF) is the fruit of *Gardenia jasminoides* Ellis and is traditionally prescribed to treat pyogenic infections and skin ulcers [9]. The GF has been included in a traditional herbal formula which is commonly used for the treatment of eczema [10]. The antiallergic effects of GF and its constituent, geniposide, in a mouse model of *Dermatophagoides farinae* extract-induced atopic dermatitis have been recently reported [11]. Moreover, GF effectively protects the keratinocytes from UV irradiation-induced cell death, indicating the potential as a topically applied material [12]. Hence, the present study was conducted to investigate the protective effects of an ethanolic extract of GF (GFE) and its components on skin disruption by diesel exhaust particulate (DEP) stimulation and to explore the mechanisms responsible.

## 2. Materials and Methods

### 2.1. Plant Material

Gardenia Fructus (GF) was purchased from Kyung Hee University Hospital (Seoul). GF (16 g) was extracted with 200 ml of EtOH at 70°C for 3 h, after which the extract was filtered through Whatman #2 filter paper (Whatman International, Maidstone, UK), evaporated on a rotary vacuum evaporator, and freeze-dried (FD8508S, Busan, Korea) (yield 13.75% w/w). The dried ethanol extract of GF obtained (GFE) was dissolved in distilled dimethyl sulfoxide and sterilized by passing it through a 0.22 *μ*m syringe filter (Millipore, MA, USA).

### 2.2. Preparation of Diesel Exhaust Particulate (DEP) Solution

Standard reference material diesel particulate matter SRM 2975 (DEP) was obtained from NIST (National Institute of Standards and Technology, Gaithersburg, MD, USA). According to the NIST certificate of analysis, the major components of this material are polycyclic aromatic hydrocarbons, such as fluoranthene, phenanthrene, chrysene, and naphthalene. DEP stock solution (10 mg/ml) was prepared in 1X PBS and sonicated for 5 min (30 s on, 15 s off; amplitude 25%). DEP solution was vortexed thoroughly before every treatment to avoid particle aggregation.

### 2.3. HPLC Analysis

**Figure 1:**
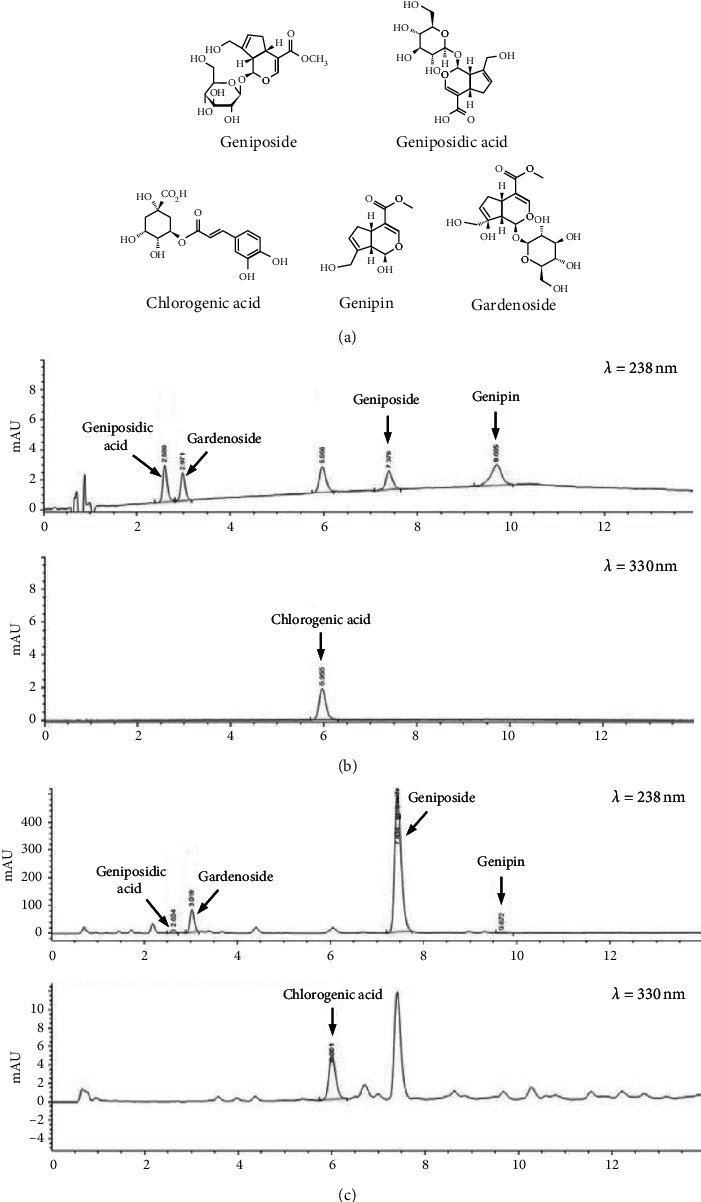
Primary components of GFE as determined by HPLC. (a) Chemical structures of geniposide, geniposidic acid, chlorogenic acid, genipin, and gardenoside. HPLC chromatograms of these primary components (b) and GFE (c).

Geniposide (CFN98261, purity 98.9%), geniposidic acid (CFN98337, 98.9%), chlorogenic acid (CFN99116, 99.5%), genipin (CFN99142, 99.6%), and gardenoside (CFN90237, 99.4%) were purchased from ChemFaces Biochemical Co. Ltd (Wuhan, China). The chemical structures of these five commercial standards are shown in Figure 1(a). Levels of these five components in GFE were determined using an HPLC 1290 system (Agilent, Santa Clara, CA, USA) at the Korea Basic Science Institute (Seoul). Extracted samples (10 *µ*l) were separated on an Extend C18 column (2.1 × 150 mm, 5 *µ*m, Agilent), which was operated at 25°C and a flow rate of 0.5 ml/min using a dilute phosphoric acid (A; 0.4%) and acetonitrile (B) gradient, as follows: 5 to 20% (B) over 20 min followed by equilibration for 5 min. Genipin, geniposidic acid, geniposide, and gardenoside were detected at a UV wavelength of 238 nm and chlorogenic acid was detected at 330 nm. Compounds in GFE were quantified using standard calibration curves prepared using the commercial standards.

### 2.4. Cell Culture and Treatments

HaCaT cells (a human keratinocyte cell line) were cultured in high glucose Dulbecco's modified Eagle's medium (LM 001-05, Welgene Inc., Gyeongsangbuk-do, Korea) supplemented with 10% heat-inactivated fetal bovine serum (TMS-013-BKR), 100 U/ml penicillin, and 100 *μ*g/ml streptomycin (#15140122) (Invitrogen, Carlsbad, CA, USA) at 37°C in a humid 5% CO2 environment. The cells were made quiescent when 70–80% confluent by starvation in serum-free medium for 24 h, and then treated with different concentrations of GFE.

### 2.5. Cell Viability

The cytotoxic effects of GFE and its five components on HaCaT cells were examined using 2,3-bis-(2-methoxy-4-nitro-5-sulphenyl)-2H-tetrazolium-5-carboxanilide (XTT) (#11465015001, Roche Diagnostics, Mannheim, Germany). Cells were treated with GFE (1, 10, or 100 *μ*g/ml) or with geniposide, geniposidic acid, chlorogenic acid, genipin, or gardenoside (1, 10, 50 *μ*M) for 24 h; then, 50 *μ*l of XTT solution was added and incubated for 4 h. Absorbances were measured at 450 nm (reference wavelength 650 nm) using a microplate reader (Molecular Devices, Sunnyvale, CA, USA).

### 2.6. Reactive Oxygen Species (ROS) Production

Percentages of cells exhibiting oxidative stress were determined using a Muse Oxidative Stress Kit (#6C3598, Millipore, Billerica, MA, USA). Briefly, 10 *μ*l of cell suspension in 1X Muse assay buffer was added to 190 *μ*l of Muse Oxidative Stress working solution and incubated in the dark for 30 min at room temperature. The analysis was then performed using a Muse Cell Analyzer (Millipore, Billerica, MA, USA).

### 2.7. Cell Apoptosis Analysis

Percentages of apoptotic cells were determined using an Annexin V & Dead Cell kit (#637362, Millipore, Billerica, MA, USA). HaCaT cells were pretreated with GFE (10, 100 *μ*g/ml) or ascorbic acid (1 mM) for 1 h and then stimulated with DEP (100 *μ*g/ml) for 24 h. 100 *μ*l of Annexin V and Dead Cell reagent and 100 *μ*l of cell suspensions were mixed in microtubes and incubated in the dark for 20 min at room temperature. Cells were analyzed using a Muse Cell Analyzer.

### 2.8. Western Blot Analysis

HaCaT cells were lysed with RIPA lysis buffer (WSE-7420, Atto, Tokyo). Proteins were then extracted by centrifuging at 8,000xg for 15 min at 4°C and collecting supernatants. Cell nuclear and cytoplasmic extracts were prepared using NE-PER extraction reagents (#78833, Thermo Fisher Scientific, Waltham, IL, USA) according to the manufacturer's protocol. Next, 25–50 *µ*g of proteins was resolved by 10% SDS-PAGE electrophoresis and transferred to polyvinylidene difluoride (PVDF) membranes (Merck Millipore, Carrigtwohill, Ireland), which were subsequently blocked with 5% skim milk in 1X PBS for 2 h at room temperature, incubated with primary antibodies and then horseradish peroxidase- (HRP-) conjugated anti-IgG antibody. Anti-Nrf2 (ab137550), anti-HO-1 (ab13243), anti-NQO-1 (ab34173), antioccludin (ab216327), and anticlaudin (ab15098) were purchased from Abcam (Cambridge, MA, USA), anti-PARP (#9542S) from Cell Signaling Technology (Danvers, MA, USA), anti-Bax (sc-493) from Santa Cruz Biotech (Paso Robles, CA, USA), anti-Bcl-2 (OP60T) from Oncogene Research Products (La Jolla, CA, USA), and anti-*β*-actin (A1978) from Sigma-Aldrich (St. Louis, MO, USA). Blots were detected by enhanced chemiluminescence (BioRad, Hercules, CA, USA) and protein band intensities were quantified using GelPro V3.1 software (Media Cybernetics, Rockville, MD, USA).

### 2.9. Animal Experiments

BALB/c (6-week-old, male) mice were purchased from Koatech Lab Animal Inc. (Seoul). All animal experimental procedures were performed in accordance with protocols approved by the Institutional Animal Care and Use Committee of Dongguk University (IACUC-2017-015). Mice were acclimated for 1 week and maintained in an environmentally controlled room (22–24°C and 50–60% RH) under a 12 h light/dark cycle with free access to food and water. Mice were randomly divided into five groups of five animals, that is, a normal control group (the NC group), a DEP treated group (the DEP group), a DEP and 1 mg/ml geniposide treated group (the G1 group), a DEP and 1 mg/ml geniposide treated group (the G10 group), and a DEP and 1 mg/ml dexamethasone treated group (the DEX group). Dorsal skins were stripped 10 times using adhesive tape to cause acute skin barrier disruption before DEP treatment. DEP was dispersed in 1X PBS, spread on a sterile pad (100 *µ*g/cm^2^), and applied every 2 days to shaved dorsal skin for 6 days to mice in the four treatment groups. After pad removal on day 7, mice were topically treated with 200 *μ*l of prescribed treatments on dorsal skin once daily for 3 days. On day 11, all mice were sacrificed and skin tissues were collected for histological and immunohistochemical analysis.

### 2.10. Histological and Immunohistochemical Examinations

Portions of dorsal skin samples were fixed in 4% paraformaldehyde and embedded in paraffin. Sections (5 *µ*m) were then cut and stained with hematoxylin and eosin (H&E). For immunohistochemical staining, sections were incubated overnight at 4°C with primary antibodies (anti-8-OHdG, anti-Bax, and antioccludin), washed, and incubated with HRP-conjugated antibodies for 1 h at room temperature. Peroxidase activities were determined using an AEC chromogen kit (AEC101, Sigma-Aldrich, St. Louis, MO, USA), and a digital camera (Olympus UC30, Japan) mounted on a phase-contrast microscope (Olympus CK40-32PH, Japan) using DIXI image solution 2.89 software (DIXI Optics, Daejeon, South Korea).

### 2.11. Statistical Analysis

The analysis was conducted using Student's t-test for unpaired experiments. Results are presented as the means ± standard deviations (SD) of at least three independent experiments, and statistical significance was accepted for *p* values < 0.05.

## 3. Results

### 3.1. Quantitative Analysis of the Chemical Constituents of GFE

Representative HPLC chromatograms of the standard compounds and GFE are shown in Figure 1. The retention times of geniposidic acid, gardenoside, chlorogenic acid, geniposide, and genipin were 2.625, 2.99, 6.013, 7.381, and 9.679 min, respectively. The concentrations of these five components in GFE ranged from 0.401 to 202.116 *µ*g/mg (Table 1).

### 3.2. Effects of GFE on ROS Production in DEP-Stimulated HaCaT Cells

**Figure 2:**
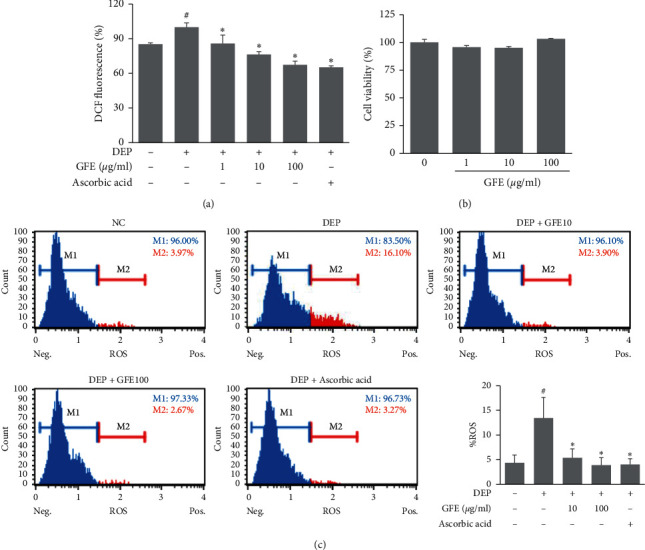
Effects of GFE on ROS production in DEP-stimulated HaCaT cells. (a) Percentages of cells exhibiting oxidative stress (ROS positive cells) were determined using an Oxidative Stress Kit. Cells were pretreated with GFE (10, 100 *μ*g/ml) for 1 h and then stimulated with DEP (100 *μ*g/ml) for 12 h. (b) Cytotoxic effects of GFE in HaCaT cells. Cells were treated with GFE (1, 10, 100 *μ*g/ml) for 24 h and cell viabilities were assessed using an XTT kit. Data are presented as mean ± SD (*n* = 3). ^#^*P* < 0.05 vs. normal controls (NC), ^*∗*^*P* < 0.05 vs. DEP treated cells.

Initially, we examine the effects of GFE on ROS production in keratinocytes. As shown in Figure 2(a), DEP markedly increased ROS levels in HaCaT cells as determined by the percentage of ROS-positive cells. However, groups pretreated with GFE showed significant decreases in ROS generation compared with the DEP-stimulated group. Moreover, GFE did not affect HaCaT cell viability, indicating inhibition of ROS production by GFE was probably not due to a cytotoxic effect (Figure 2(b)).

### 3.3. Effects of GFE on the Antioxidant Pathway in HaCaT Cells

**Figure 3:**
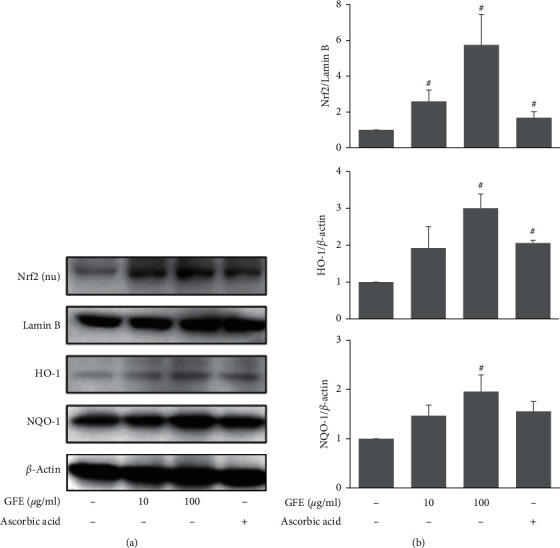
Effects of GFE on the antioxidant pathway in HaCaT cells. (a) Cells were treated with GFE (10, 100 *μ*g/ml) for 30 min (to measure Nrf2 expression) or for 24 h (to measure HO-1 and NQO-1 expressions). Expressions of Nrf2, HO-1, and NQO-1 were determined by western blot. (b) Densitometry analysis of the expressions of Nrf2, HO-1, and NQO-1. Data are presented as mean ± SD (*n* = 3). ^#^*P* < 0.05 vs. normal controls.

To understand the underlying mechanism, we investigated the effect of GFE on the transcription factor, Nrf2, which is responsible for initiating antioxidant pathways to eliminate ROS. Treatment with GFE (100 *μ*g/ml) showed an increase in the nuclear translocation of Nrf2 and expression of antioxidant response protein HO-1 and NQO-1 in HaCaT cells (Figure 3).

### 3.4. Effects of GFE on Apoptosis in DEP-Stimulated HaCaT Cells

**Figure 4:**
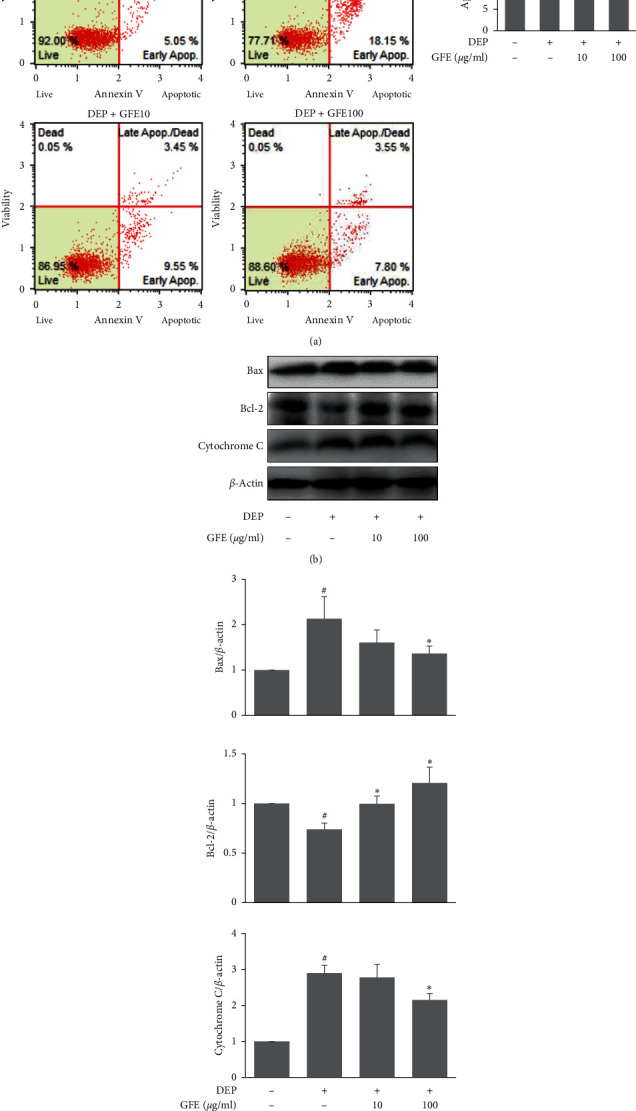
Effects of GFE on apoptosis in DEP-stimulated HaCaT cells. (a) Percentages of apoptotic cells were determined using an Annexin V & Dead Cell kit. Cells were pretreated with GFE (10, 100 *μ*g/ml) for 1 h and then stimulated with DEP (100 *μ*g/ml) for 24 h. (b) The expressions of Bax, Bcl-2, and cytochrome C were determined by western blot. (c) Densitometry analysis of Bax, Bcl-2, and cytochrome C expression. Data are presented as mean ± SD (*n* = 3). ^#^*P* < 0.05 vs. normal controls (NC), ^*∗*^*P* < 0.05 vs. DEP treated cells.

ROS act as a central second-messenger in apoptotic signaling pathways. Previous reports have also suggested that DEP-induced oxidative stress could result in keratinocyte apoptosis [13]. DEP increased percentages of apoptotic cells determined by Annexin V staining (Figure 4(a)) and significantly increased the expressions of Bax and cytochrome C but decreased the expression of Bcl-2. However, GFE pretreatment prevented DEP-induced keratinocyte apoptosis by modulating Bax and Bcl-2 levels and the ROS/Cytochrome C pathway. More specifically, pretreatment with GFE at 100 *μ*g/ml increased the expression of Bcl-2 and decreased the expressions of Bax and cytochrome C (Figures 4(b) and 4(c)).

### 3.5. Effects of GFE on Tight Junction Protein Expression in DEP-Stimulated HaCaT Cells

**Figure 5:**
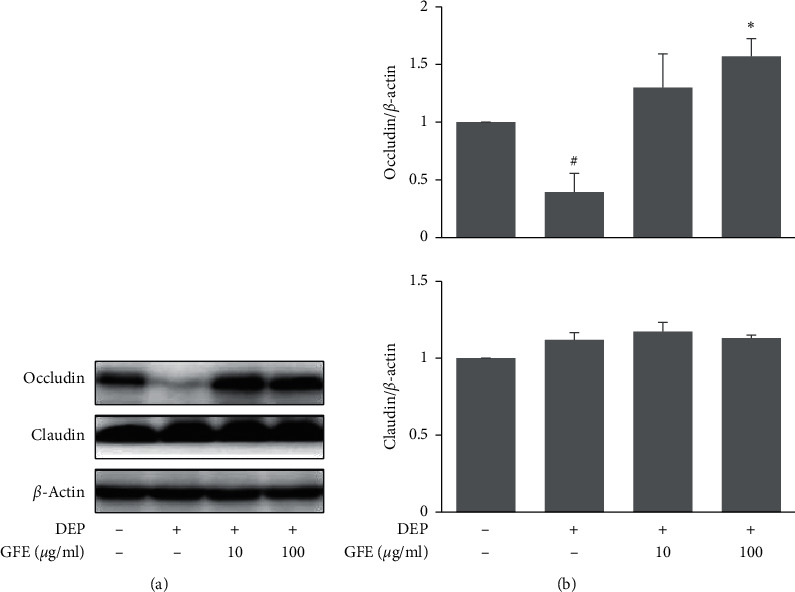
Effects of GFE on tight junction protein expressions in DEP-stimulated HaCaT cells. (a) Cells were pretreated with GFE (10, 100 *μ*g/ml) or ascorbic acid (1 mM) for 1 h and then stimulated with DEP (100 *μ*g/ml) for 24 h. The expressions of occludin and claudin were determined by western blot. (b) Densitometry analysis of occludin and claudin expression. Data are presented as mean ± SD (*n* = 3). ^#^*P* < 0.05 vs. normal controls, ^*∗*^*P* < 0.05 vs. DEP treated cells.

Tight junctions create fusion points between keratinocytes and form a barrier that protects the body from the external environment that plays an important role in dermal absorption [14]. In addition to increasing HaCaT apoptosis, DEP also reduced the expression of occludin (a tight junction protein). Subsequently, we found pretreatment with GFE at 10 and 100 *μ*g/ml increased occludin expression in DEP-stimulated HaCaT cells (Figure 5).

**Figure 6:**
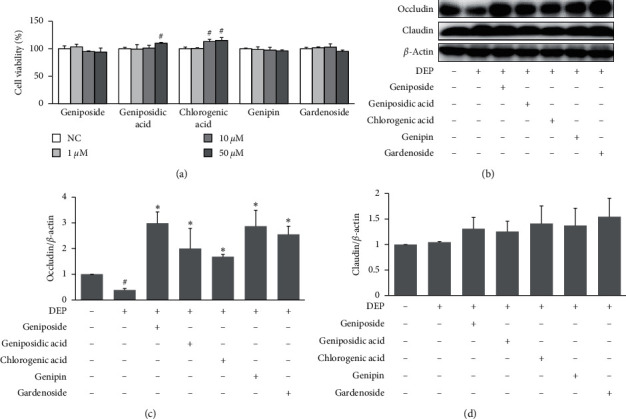
Effects of the five compounds found in GFE on tight junction protein expressions in DEP-stimulated HaCaT cells. (a) Cells were pretreated with geniposide, geniposidic acid, chlorogenic acid, genipin, or gardenoside (1, 10, 50 *μ*M) for 24 h. Cell viabilities were assessed using an XTT kit. (b) Cells were pretreated with geniposide, geniposidic acid, chlorogenic acid, genipin, or gardenoside (50 *μ*M) for 1 h and then stimulated with DEP (100 *μ*g/ml) for 24 h. The expressions of occludin and claudin were determined by western blot. (c) Densitometry analysis of occludin and claudin expression. Data are presented as mean ± SD (*n* = 3). ^#^*P* < 0.05 vs. normal controls (NC), ^*∗*^*P* < 0.05 vs. DEP treated cells.

Next, we sought to determine whether any of the five compounds detected in GFE contributed to its effects in DEP-stimulated HaCaT cells. As shown in Figure 6(a), cell viability was not affected by any of these compounds at concentrations up to 50 *μ*M. Furthermore, pretreatments with geniposide, geniposidic acid, chlorogenic acid, genipin, or gardenoside (all at 50 *μ*M) significantly increased occludin expression as compared with DEP alone (Figures 6(b) and 6(c)).

### 3.6. Effects of Geniposide on Skin in DEP-Stimulated Mice

**Figure 7:**
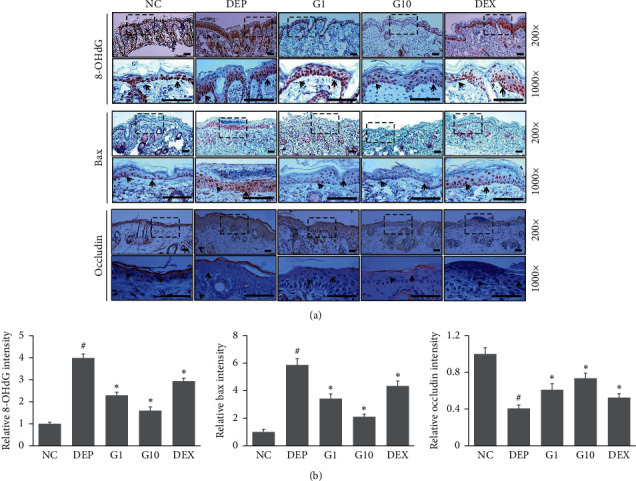
Effects of geniposide on DEP-stimulated mouse skin. DEP (100 *µ*g/cm^2^) was applied every 2 days to dorsal skin for 6 days. On day 7, mice were topically treated with geniposide (1 mg/ml or 10 mg/ml, 200 *μ*l) or DEX (1 mg/ml, 200 *μ*l) or vehicle once daily for 3 days. Immunohistochemical analysis of 8-OHdG, Bax, and occludin in dorsal skin. (a) Representative images of each group. Scale bar: 50 *μ*m. (b) Quantitative analysis of 8-OHdG, Bax, and occludin immunohistochemistry. Data are presented as mean ± SD (*n* = 3). ^#^*P* < 0.05 vs. NC group, ^*∗*^*P* < 0.05 vs. DEP group.

We further investigated the effect of geniposide on DEP-induced skin damage by immunohistochemical analysis in BALB/c mice. To determine whether geniposide protects against DEP-induced oxidative stress and DNA damage, we measured 8-OHdG expression in skin samples. 8-OHdG expression was significantly increased in skin exposed to DEP, and topical treatment with geniposide reduced this increase in 8-OHdG expression. In addition, geniposide significantly reduced DEP-induced increases in Bax expression. Moreover, the downregulation of occludin expression in DEP-stimulated mice was recovered with geniposide topical application (Figure 7).

## 4. Discussion

Oxidative stress caused by the excessive generation of ROS is a critical event in tissues exposed to DEP and damages DNA and proteins, which can lead to apoptosis and tissue destruction [15]. The present study shows DEP dose-dependently increased intracellular ROS synthesis in HaCaT keratinocytes and that GFE inhibited this DEP-induced ROS generation and increased nuclear localization of Nrf2 and upregulated the expressions of antioxidant enzymes like HO-1.

In fact, HO-1 may represent the most critical cytoprotective mechanism activated by stress, and its upregulation has been shown to decrease cellular heme levels and increase reduced glutathione levels, which shifts redox balance toward a reduced state and reduces O_2_^−^ formation. GF was reported to have antioxidant effects in another study, in which GF increased the activity of superoxide dismutase (an antioxidant enzyme) and suppressed oxidative stress in cortex and hippocampus [16].

In a previous study, ROS increased proapoptotic proteins and decreased the expressions of antiapoptotic proteins [17]. Proapoptotic Bax and antiapoptotic Bcl-2 regulate the mitochondria-mediated apoptotic pathway, particularly cytochrome C release. Recent studies have reported DEP triggers apoptosis by the upregulating Bax [13]. In the present study, DEP increased intracellular ROS levels and increased the Bax/Bcl-2 protein ratios, which suggested the mitochondria-mediated pathway is involved in DEP-induced apoptosis in HaCaT cells. On the other hand, GFE pretreatment significantly reduced DEP-induced increases in Bax/Bcl-2 ratios and cytochrome C levels. Furthermore, when mouse skins were topically treated with geniposide and DEP, geniposide decreased DEP-induced 8-OHdG (a marker of oxidative DNA damage) and proapoptotic Bax expressions. Interestingly, DEP, which is composed of atmospheric particulate matter, causes oxidative stress and cell damage through pathways without causing inflammatory response [18]. Our observations indicate the protective effect of GFE is due to the suppression of ROS-induced apoptosis.

Tight junctions (TJs) are cell-cell junctions formed by claudins, occludin, zonula occludens, and junctional adhesion molecules. In the stratum granulosum of epidermis, tight junctions seal spaces between individual cells and create a paracellular barrier [14]. Furthermore, alteration of occludin has been associated with psoriasis and atopic dermatitis, which suggests it plays an important role in skin barrier function [14]. Transmission electron microscopy studies have shown airborne particulate matter can penetrate skin tissues [19], and DEP has been reported to disrupt tight junctions and exacerbate various types of dermatitis [7]. In the present study, DEP stimulation reduced occludin expression in HaCaT cells, and pretreatments with GFE or geniposide prevented this DEP-induced reduction. Since oxidative stress induces tight junction disruption, these findings suggest that the protective effect of GFE on occludin expression is mediated, at least in part, by inhibiting ROS production. Moreover, in another study, N-acetyl-cysteine reduced ROS generation in epithelial cells and attenuated tight junction protein degradation [20].

We also found geniposide significantly increased occludin expression and protected against DEP-induced oxidative stress and DNA damage. The amount of geniposide in GFE was 202.116 *μ*g/mg and, thus, was present in GFE at considerably higher levels than the other four components. Geniposide is a hydrophilic iridoid glucoside and has been reported to suppress UV-B-induced ROS, suggesting it has an antiphotoaging effect and therapeutic potential for skin repair and regeneration [21]. Geniposide is also present in Rubiaceae family, which is one of the largest angiosperm families, and hence is an attractive proposition for the development of plant-derived cosmetics or drugs [22]. Furthermore, its high stability and skin absorption characteristics suggest it has considerable therapeutic potential for the treatment of DEP-induced skin damage [22, 23].

**Figure 8:**
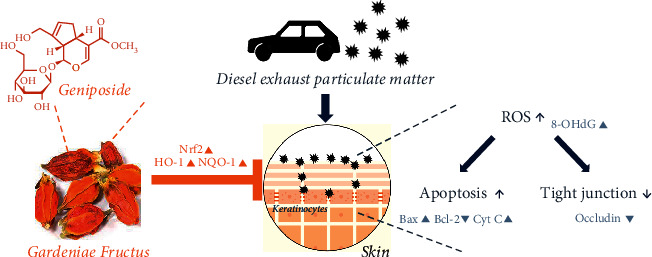
Schematic diagram showing possible mechanisms responsible for the pharmacological efficacy of GFE and its principal component geniposide against DEP exposure.

The present study has several limitations. First, mouse skin has a higher density of follicles than human skin, and this may have resulted in greater DEP penetration than is the case for human skin [24]. Second, the study was performed using a standard reference material and not authentic air pollution particles. Third, the method used to expose skin to DEP using pads obviously differed from atmospheric exposure. Nevertheless, the study shows GFE has antipollution effects and identified the mechanism responsible for its effects (Figure 8).

## 5. Conclusion

DEP-induced ROS production can lead to the activation of apoptotic processes and disrupts tight junctions in keratinocytes. GFE and its constituent geniposide both effectively protected keratinocytes against DEP-induced oxidative stress and Bax upregulation and increased occludin expression. We suggest GFE and geniposide be considered for pharmaceutical and cosmetic products that selectively protect against the effects of DEP.

## Figures and Tables

**Table 1:** Quantification of chemical constituents in GFE by HPLC.

Compound	Amount (*μ*g/mg)
Geniposide	202.116
Geniposidic acid	2.575
Chlorogenic acid	1.333
Genipin	0.401
Gardenoside	20.961

## Data Availability

The data used to support the findings of this study are included within the article.

## Abbreviations

GF: Gardeniae Fructus
DEP: Diesel exhaust particulate matter
ROS: Reactive oxygen species
Nrf2: Nuclear factor E2-related factor 2
HO-1: Heme oxygenase-1
NQO-1: NAD(P)H quinone dehydrogenase 1
TJ: Tight junction.
